# Exploring the factors that affect the transition from student to health professional: an Integrative review

**DOI:** 10.1186/s12909-021-02978-0

**Published:** 2021-11-02

**Authors:** Eric Nkansah Opoku, Lee-Ann Jacobs-Nzuzi Khuabi, Lana Van Niekerk

**Affiliations:** 1 Division of Occupational Therapy, Faculty of Medicine and Health Sciences, Stellenbosch University, Francie Van Zijl Dr. Tygerberg Medical Campus, Cape Town, South Africa; 2 Present Address: Department of Occupational Therapy, College of Health Sciences, University of Ghana, Accra, Ghana; 3 Department of Occupational Therapy and Human Nutrition and Dietetics, School of Health & Life Sciences; Glasgow Caledonian University, Cowcaddens Road, Glasgow, UK

**Keywords:** Role transition, Clinical practice, New clinicians, Professional competence, Novice professionals, Professional practice, Systematic literature review

## Abstract

**Background:**

The nature of a new health professional’s transition from student to health professional is a significant determinant of the ease or difficulty of the journey to professional competence. The integrative review will explore the extent of literature on the factors that impact the transition of new health professionals into practice, identify possible gaps and synthesise findings which will inform further research. The aim was to identify research conducted in the last two decades on the barriers, facilitators and coping strategies employed by new health professionals during their transition into practice.

**Methods:**

Whittemore and Knafl’s methodological framework for conducting integrative reviews was used to guide this review. Sources between 1999 and 2019 were gathered using EBSCOhost (including CINAHL, Medline, Academic Search Premier, Health Science: Nursing and Academic Edition), PubMed, Scopus, Cochrane and Web of Science, as well as hand searching and follow-up of bibliographies followed. The Covidence platform was used to manage the project. All studies were screened against a predetermined selection criteria. Relevant data was extracted from included sources and analysed using thematic analysis approach.

**Results:**

Of the 562 studies identified, relevant data was extracted from 24 studies that met the inclusion criteria, and analysed to form this review. Thematic analysis approach was used to categorise the findings into theme areas. Four overarching themes emerged namely: systems and structures, personal capacities, professional competence and mediating processes. Each theme revealed the barriers, facilitators and coping strategies of transition into practice among new health graduates.

**Conclusion:**

The transition into practice for new health practitioners has been described as complex and a period of great stress. Increasing clinical and practical experiences during education are required to support new health professionals in the process of closing the gap between learning and practice. Continued professional development activities should be readily available and attendance of these encouraged.

## Background

The transition into practice for new practitioners has been described as complex and a period of great stress [1, 2]. The academic environment and the practice environment have been described as different worlds as knowledge acquired in the classroom was deemed practically untransferable to the real world [3, 4]. Due to the gap between academic and practice contexts, evidence suggests that new health professionals might be overwhelmed with feelings of inadequacy [5], unpreparedness [6] and doubtfulness related to their competence [4, 7]. Evidence also suggests that, the reality of practice is experienced as a shock by new health practitioners [6, 7]; feelings that might negatively affect- the personal and professional confidence of new health professionals [8]. Other challenges experienced during the transition included role confusion [9], overwhelming workloads [3], sophisticated workplace protocols [10] and lack of respect and recognition [11].

The nature of a new health professional’s transition from student to health professional has been shown to be a significant determinant of the ease or difficulty of his/her journey to professional competence [2, 12]. Several strategies were found to alleviate the challenges that characterise the transition from student to health professional. Consistent emphasis is placed on supervision to help new health professionals relate the knowledge acquired in the classroom to practice [4, 12–15]. Effective supervision equips new health professionals with skills needed to function in their respective areas of practice [13]. Moores and Fitzgerald [12] established that meaningful interactions with other new health professionals in the form of study groups, peer support meetings and social interaction sessions contribute significantly to successful transitions to practice [14]. Other support strategies emphasised in the literature include adequate orientation for new health professionals [11, 16], support from more experienced senior colleagues [15], preceptorship programmes [17] and other health professionals [18]. New health professionals have also been advised to utilise continuing education opportunities. Evidence suggests that continued professional development avenues positively impact on new health professionals’ self-confidence and professional identity [7].

This integrative review aimed to identify research conducted in the last two decades (1999-2019) on the barriers and facilitators associated with new health professionals’ transition into practice and the coping strategies employed to ensure successful transition into practice

## Methods

**Fig. 1:**
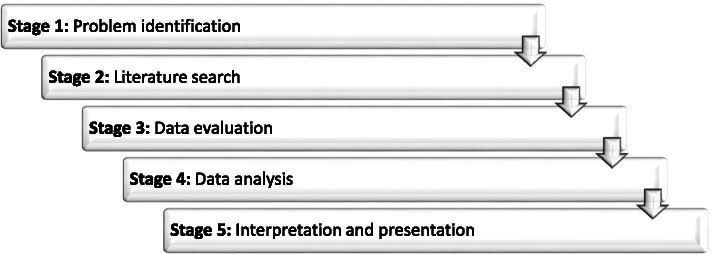
Stages of Whittemore and Knafl’s Methodological Framework [20]

The integrative review commenced with publication of the protocol so as to obtain peer input [19]. Whittemore and Knafl’s [20] methodological framework for conducting integrative reviews guided this review (See Fig. 1); the process will now be discussed in detail.

### Problem Identification (Preparing guiding question)

The overarching question that guided this review was ‘what factors affect the transition of new health professionals from students to health professionals?’ To capture the scope and the diversity of available literature, three specific research questions were developed to answer the question.

- What challenges do new health professionals face during transition into practice?

b. What factors facilitate the transition of new health professionals into practice?
c. What coping strategies do new health professionals employ to ensure successful transition into practice?

### Literature search

A search was done to identify literature from five electronic databases namely PubMed, EBSCOhost (including CINAHL, Medline, Health Science: Nursing and Academic edition), Scopus, Cochrane and Web of Science. The first search was done April 3, 2019. The search strategy included the keywords New clinician OR Novice professional OR Health student AND Transition* AND Clinical practice AND Clinical competence OR Professional Competence. The search strategy used was developed in consultation with an experienced subject librarian. Limiters applied were published date (January 1999 to April 2019), SmartText searching and Language (English only). Hand searches and follow up of the reference lists of the included articles was done to retrieve literature that were not found in the databases.

### Data evaluation

Titles and abstracts of all retrieved sources were uploaded onto the Covidence Platform, which was used to manage the project. The Covidence platform automatically removed duplicates before the review process began. Quality assessment was undertaken to ensure the clarity of study aim, the participants and the relevance of the of the study to answer the research question. All studies were assessed for eligibility by three independent reviewers according to the criteria contained in Table 1.

**Table 1:** Selection criteria

Inclusion criteria	Exclusion criteria
Peer-reviewed research source on new health professionals’ transition from students to health professionals.	Conference abstracts, doctoral theses and grey literature.
Primary sources of systematic reviews that meet the inclusion criteria.	Systematic or literature reviews
Published in English	
Published after 1999	

Following the title and abstract screening, full texts of the included studies were uploaded for full text screening against the same predetermined selection criteria. Conflicts were resolved through consultation among the three reviewers until consensus was reached. Once consensus was reached on the eligibility of sources, data was extracted from full text publications using a data charting form adapted from Uys et al [21]. For each of the included studies, the researcher extracted the study characteristics (author names, publishing journal, year study was published, country of study and the population of the study), study aims, objectives and/or research questions, the study design and the findings (with particular emphasis on the barriers, the facilitators and coping strategies of transition into practice).

### Data analysis and presentation

Once the data extraction process was completed, findings were analysed and categorised into themes areas using a thematic analysis approach [22]; this involved summary and categorization of data into codes, sub-themes and main themes. The analysis focused on extracting data that met the objectives of this review. The first author did the analysis, which was then reviewed and refined with the assistance of the second and third authors. Once the data evaluation and analysis processes were completed, the review findings were presented in the form of descriptions and narrations.

## Results

### Study selection

**Fig. 2:**
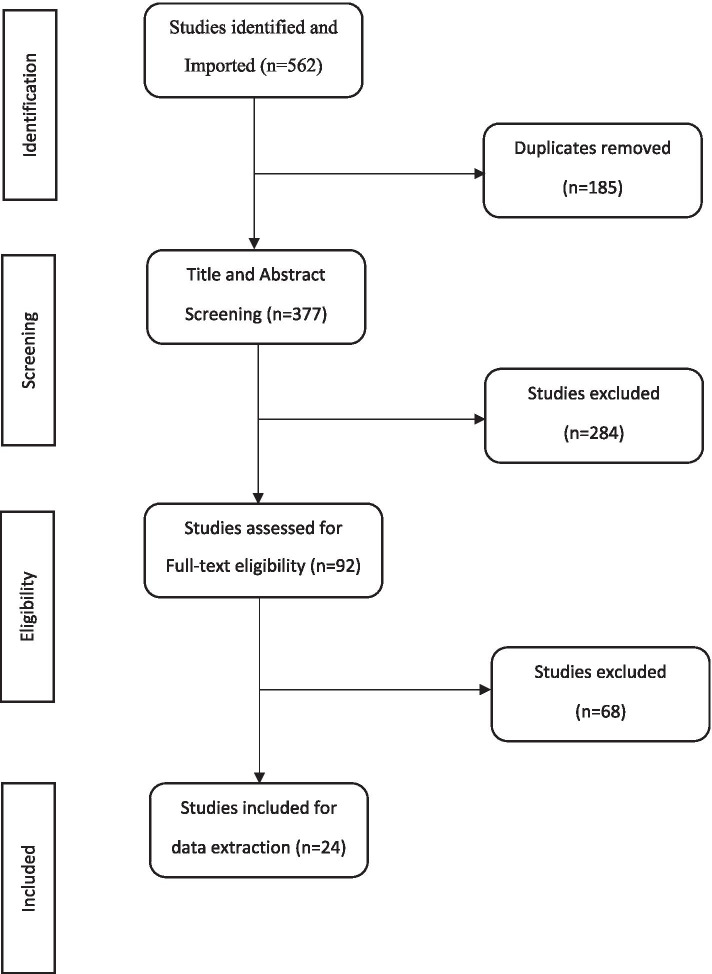
PRISMA flow chart of study selection

The primary search strategy identified 562 studies from which 185 duplicates were removed. The title and abstract of 377 sources were screened and 284 were excluded. The full texts of the included sources were uploaded onto the Covidence platform for full text screening against the same predetermined inclusion and exclusion criteria. Overall, 92 studies were assessed for full-text eligibility. A final total of 24 studies met the inclusion criteria and relevant data was extracted and analysed to form this review (See Fig. 2: PRISMA flow chart of study selection)

### Study characteristics

Most sources originated in Australia (*n* = 8; 33.3%), followed by Canada (*n* = 5; 20.8%), the USA (*n* = 3; 12.5%), the UK (*n* = 2; 8.3%) and Norway, Turkey, Oman, Jordan, Philippines and Ireland (*n*=1 for each country; 4.2%). Twenty sources used qualitative methodologies, 3 used quantitative methodologies and one study used mixed methods. Thirteen sources pertained the profession of nursing (54.2%), seven were on occupational therapy (29.2%), two about medicine/medical doctors (8.3%) and one each on physiotherapy and midwifery (4.2%). Most studies pertaining physiotherapists, midwives and doctors, focused on the transition from student to forms of practice that precede independent practice, for example clinical placements, internships and residencies. These did not form part of this review thus accounting for the low number of sources for these professions. Table 2 presents a summary of the sources that were included in the review including characteristics such as first author, year of publication, country of origin, study aims, sample size, profession of participants, the methodology used and the publishing journal.

**Table 2:** Summary of sources included in the final analysis

First author (Ref), year, country of origin	Study aims/objectives/question	Sample size, profession	Methodological tradition, publishing journal
1. O'shea [23], 2007, Ireland (High Income)	To explore newly qualified staff nurses’ experiences of being on clinical placement post registration, and the meanings that this experience held for them.	*n*=10 Newly qualified nurses,	Qualitative phenomenology, Clinical Nursing
2. Labrague [3], 2019, Philipine (Lower-middle income)	This study describes the transition experiences of newly graduated Filipino nurses during their initial clinical placement process one-year post registration	*n*=15 newly graduated nurses,	Qualitative phenomenology, Nursing Forum
3. AbuAlRub [24], 2018, Jordan (Upper-middle income)	To explore the challenges that face new Jordanian nurses in the first year of employment	*n*=30, Newly qualified nurses, and *n*=6 key informants	Qualitative phenomenology, International Nursing Review
4. Awaisi [17], 2015, Oman (High income)	To explore the experiences of new clinician nurses during their transition period in the Sultanate of Oman	2009 (*n*=15) and 2010 (*n*=10) cohorts of nursing clinicians, students (*n*=8), preceptors (*n*=6), clinical instructors (*n*=5), head nurses (*n*=5), and managers (*n*=4) who had experienced working with new clinician nurses.	Qualitative case study International Journal of Nursing Studies
5. Black [25], 2010, USA (High Income)	To explore the experiences, learning, and development of promising novice therapists throughout their first year of practice in the United States.	*n*=12, Novice physical therapist	Grounded theory, Journal of the American Physical Therapy association
6. Brennan [26], 2010. UK (High income)	To explore the experiences of junior doctors during their first year of clinical practice.	*n*=31, Newly qualified doctor,	Qualitative phenomenology, Medical Education
7. Casey [27], 2004, USA (High Income)	To identify the stresses and challenges experienced by cohorts of clinician nurses.	*n*=270, New clinician nurses	Quantitative cross-sectional survey. Nursing Administration
8. Clare [5], 2003, Australia (High income)	To explore the transition from undergraduate student to clinician nurse.	*N*=550, Clinician nurses and *N*=350 directors of nursing.	Mixed methods Collegian
9. De Bellis [4], 2015, Australia (High Income)	To identify the issues and difficulties experienced by new nursing clinicians in the clinical environment as they commence their career as registered nurses	*n*=21 Newly registered nurses.	Qualitative phenomenology, Contemporary Nurse
10. Doherty [10], 2009, Australia (High Income)	To investigate the perception of new graduates on their preparation for practice, at seven months post-graduation.	*n*=18 Newly registered first cohort of occupational therapists.	Quantitative cross-sectional survey, Australian Occupational Therapy Journal
11. Tryssenaar [6], 2001, Canada (High income)	The study explored the lived experience of rehabilitation students during their final placement and first year of practice.	*n*=6 Occupational therapist (*n*=3), and 3 Physical Therapy (*n*=3) students	Qualitative phenomenology, American Journal of Occupational Therapy
12. Lee [18], 2003, Australia (High Income)	To explore the experiences of five new clinician occupational therapists.	*n*=5 New occupational therapy clinicians	Qualitative phenomenology, Australian Journal of Rural Health
13. Mangone [28], 2016, Australia (High income)	To explore the effectiveness of addressing transitional issues during debriefing sessions for new clinician nurses and trainee enrolled nurses.	Three focus groups consisting of four to five new nursing graduates.	Qualitative study, Contemporary Nurse
14. Nour [29], 2018, Canada (High income)	To explore the experiences of newly graduated- nurses in acute healthcare settings within Canada.	*n*=14 Newly registered nurses	Grounded theory, Canadian Journal of Nursing Research.
15. Phillips [11], 2014, Australia (High income)	To identify the factors that clinician nurses believe assists them in successful transition to registered nurse practice.	Already existing 8 focus group interviews (*n* = 67) and participant responses to opened-ended questions from an electronic survey (*n* = 392)	Qualitative study. Nurse Education in Practice
16. Regan [15], 2017, Canada (High income)	To describe new clinician nurses’ transition experiences from the perspectives of new clinician nurses and nurse leaders in unit level roles.	*N* = 42 New clinician nurses and nurse leaders (*n*=28)	Qualitative phenomenology, Nursing Management
17. Reynolds [30], 2014, UK (High income)	To elicit the lived experience of newly qualified midwives from the point of registration to 12 months post-registration	*n* = 12 Newly qualified midwives,	Qualitative phenomenology, British Journal of Midwifery
18. Seah [7], 2011, Australia (High income)	To explore the lived experience of transition from student to practitioner within first six months of practice.	*n* = 8 Newly graduated cohort of Master of Occupational Therapy clinicians	Qualitative phenomenology, Australian Occupational Therapy Journal
19. Tastan [31], 2013, Turkey (Upper-middle income)	To identify the factors affecting the transition period of newly graduated nurses.	*n* = 234 Newly graduated nurses	Descriptive quantitative, International Nursing Review
20. Toal-Sullivan [32] 2006, Canada (High income)	To explore the experience of transition and how beginning practitioners learn about doing practice.	*n* = 6 Newly graduated Occupational therapists,	Qualitative phenomenology, British Journal of Occupational Therapy
21. Tryssenaar [33] 2001, Canada (high income)	To describe the lived experience of becoming an occupational therapist.	*n*=1 Newly graduated Occupational therapist, case study	Qualitative Phenomenology British Journal of Occupational Therapy
22. Wangensteen [34] 2007, Norway (High income)	To illuminate how recently graduated nurses’ experience their first year as a nurse.	*n* = 12 Recently graduated nurses	Qualitative Phenomenology, Journal of Clinical Nursing
23. Zinsmeiter [35] 2009, USA (High income)	To gain insight into the transition period of clinician nurses.	*n* = 9 Newly graduated nurses,	Qualitative Phenomenology, Journal of Nursing in Staff Development
24. Bearman [36] 2011, Australia (High income)	To explore the experiences of developing professional identities during housemanship	*n*=30 New graduated medical doctors, and intern supervisors (*n*=6)	Grounded theory, Advancement in Health Science Education

**Fig. 3:**
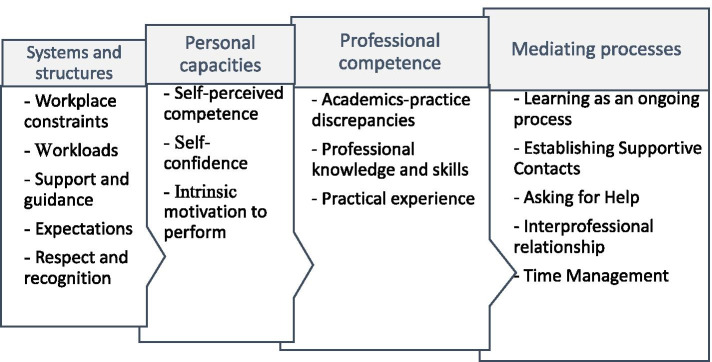
Themes and sub-themes derived from data analysis

Data extraction was done with the three research questions in mind. Multiple factors which affected the multifaceted experiences of new health professionals during their transitions, either positively or negatively, were identified. These factors pertained experiences of new health professionals with self, clients, other health professionals, workplace protocols and the healthcare delivery system as a whole. Once data were charted, findings were summarised and categorised into codes, sub-themes and main themes. Four overarching themes were developed: ‘*systems and structures’, ‘personal capacities’, ‘professional competence’ and ‘mediating processes’* with a number of sub-themes (see Fig. 3: *Theme and sub-themes derived from data analysis*).

## Discussion

The challenges, facilitators and the coping strategies shown to affect the transition into practice for new graduates from various health professions will now be discussed in more detail.

### Theme 1: Systems and structures

The theme ‘*systems and structures’* reflects the barriers new health practitioners had to overcome and the challenges they faced during transition to practice; it comprises five sub-themes, namely *workplace constraints, workloads, support and guidance, expectations, and respect and recognition.*

#### Workplace constraints

Among the challenges encountered by new health professionals were those relating to the complexity of systems in the workplace. New health professionals reported having a naive understanding of the hierarchy of the system, administrative processes, workplace politics and organisational dynamics; this impacted on their transition into practice [4, 6, 14, 16, 33]. One study reported that, not knowing the “what, how, why, where and when” of workplace routines posed various challenges for new health professionals [4]. Variations in operations and administration also served as a source of frustration for new health professionals as they moved between workplaces [36]. New health professionals were expected to automatically adapt to ‘the-way-things-are-done’ and the ‘it-is-always-done-this-way’ operational culture in the wards [15]. Many of these procedures were experienced as contrary to what new health professionals had been taught, thus causing confusion [17].

#### Workloads

Complex and overwhelming work-related responsibilities were experienced among new health professionals [3, 4, 16, 29, 31]. One study reported that new health professionals were expected to handle complex cases and procedures which they considered unreasonably beyond their capabilities as novice professionals [3]. Research also reported heavy patient loads among new health professionals which require them to either work overtime or work under pressure in order to meet all responsibilities [32]. New health professionals generally felt overworked at the end of the day [28].

#### Respect and recognition

A lack of respect and recognition for new health professionals during transition into practice was reported. Phillips et al [11] suggested that new health professionals were not afforded the respect they deserved, especially those that were younger. They emphasized that, lack of respect undermined new health professionals’ self-confidence, which translated into a lack of self-worth [11]. Unprofessional behaviour from other health professionals or senior colleagues, included experiences of being treated as subordinates, [17] bullying and insults [5], impacted negatively on the adjustment of new health professionals. Reynold et al [30] and Tryssenaar [33] reported that new health professionals did not feel valued in their practice. In fact, one study reported that new health professionals considered quitting their jobs after the first year due to lack of recognition and appreciation and an overall experience of dissatisfaction at work [29].

#### Support and guidance

The importance of having a well-structured system of support and guidance for new health professionals during their transition was emphasized. New health professionals who received sufficient orientation reported doing well during their transition into practice [11, 13, 16, 31, 32]. Conversely, new health professionals who did not get sufficient orientation encountered difficulties with the transition [4, 16, 21, 26]. In addition to orientation programmes, strategies found to support new health professionals’ transition into practice included residency programmes [24], preceptorship programmes [13, 28, 31] and mentoring programmes [5, 16, 22]. New health professionals needed support from experienced senior colleagues [4, 5, 11, 16, 23, 30, 31] as well as peers [4]. New health professionals reported feeling motivated to perform better when they received feedback on their performance from other health professionals [34] and clients [32]. Particular emphasis was also placed on supervision as an effective strategy to help new health professionals overcome the stressors of the transition [6, 7, 11, 13].

#### Expectations

New health professionals reported feeling overwhelmed by unrealistically high expectations placed on them [3, 13, 25, 26, 29]. Labrague et al [3] reported that new health professionals felt pressured and stressed when unachievable expectations were placed on them. Nurses in Clare & Loon’s [5] study expressed gratitude to their superiors for having realistic expectations of their skills. They further emphasized that realistic expectations gave them the opportunity to grow their confidence [5]. Furthermore, new health professionals reported that they were ignorant of what was expected of them [7, 26]. In Zinsmeiter’s [35] study, new health professionals reported that when all health professionals (new and existing) have clear expectations of their role, the transition becomes comfortable.

### Theme 2: personal capacities

In the theme “personal capacities” the personal characteristics of new health professionals that influence their transition from student to health professional were captured; it comprised three sub-themes, namely *self-perceived competence, self-confidence,* and *intrinsic motivation to perform.*

#### Self-perceived competence

One factor that affected new health professionals’ transition into practice was their perception of their own competence. New health professionals’ perception that they do not know enough made them question their competence and readiness for practice [4, 6, 7, 18–22, 26, 27, 31–33]. Several situations were reported where new health professionals were caught in a dilemma related to diagnosis, assessment or treatment procedures [5, 7, 23]. Nurses in Clare’s [5] study described the overwhelming feeling of inadequacy as the worst aspect of their transition. Feeling inadequate resulted in new health professionals feeling vulnerable and fearful of taking on responsibilities because of their fear of making mistakes [23, 29].

#### Self-confidence

Confidence was emphasized as a personal quality that contributes significantly to the success of transition into practice. However, self-confidence seemed to be determined primarily by new health professionals’ perception of how competent they were and how prepared they were for practice [4, 10, 11, 17, 21, 22, 24, 32]. A quantitative cross-sectional survey by Doherty et al [10] revealed that new occupational therapy graduates’ perceived self-confidence had a significant positive association with their self-perceived competence level in clinical decision making.

#### Intrinsic motivation to perform

New health professionals reported several factors motivating them to continue to pursue competence in the face of challenges encountered during transition. The fact that they were playing an integral role of changing the health of patients for the better motivated them to persist. Others found motivation by associating their role to the spiritual benefits they expected in future [17]. Other new health professionals were motivated by the excitement in acquiring new skills and growing in their professions [3, 5].

### Theme 3: Professional competences

This theme reflected the relationship between knowledge, skills and attitudes new health professionals acquired through their education, and practising in the field. Three sub-themes emerged, namely *academics-practice disparity, professional knowledge and skills, and practical experiences.*

#### Academics-practice disparity

Research findings reported a dichotomy between what was learnt in the classroom and the expectations of actual performance in practice [3, 4, 16, 21, 29]. De Bellis et al [4] emphasized that the knowledge participants in their study acquired from their undergraduate education was not applicable in their practice. The incongruency between education and practice was believed to often lead to a *reality shock* in the practice environment [24]. The sources reviewed suggested that new health professionals experienced high levels of tension, coupled with anxiety and nervousness upon entering the world of practice [6, 7, 20, 25–27, 29, 32, 33]. O’shea et al. [23] emphasized that the ‘reality shock’ and anxiety among new health professionals was intense, particularly in the first five months of transition into practice. New health professionals often experienced disconnect between their expectations of practice and the reality of practice [32]. They experienced varying levels of stress beyond their expectations which impacted their transition [3, 6, 13, 20, 21, 25, 26, 30–33]. New health professionals often felt incapable of managing stressful emotional work-related situations such as death and dying [13, 20, 23].

#### Professional knowledge and skills

The sources reviewed suggested that it is in practice that new health professionals become aware of deficits in their knowledge and skills. The gap between education and practice can cause a mismatch between new health professionals’ expectations of their roles and what is actually practiced in the field, leading to role confusion [6, 13, 29]. New health professionals demonstrated inadequacy in clinical practice skills such as communication skills [10, 22, 24], organisational and management skills [16, 20, 22], clinical decision-making skills [5, 22] and skills required for specific practice areas [6, 16, 29]. Newly qualified occupational therapists in a study by Toal-Sullivan [32] reported that they felt unprepared in specialised clinical skills, such as splinting, cognitive remediation, wheelchair prescription, hand therapy and home safety equipment.

#### Practical experience

Inadequacies in the knowledge and skills of new health professionals was strongly associated with insufficient practical and clinical exposure in their undergraduate training [4–6, 21]. Increasing hands-on experience of new health professionals during education can help prevent inadequate knowledge and skills during practice [7]. Occupational therapists in their first year of practice emphasized that prior clinical placement experience helped ameliorate the stress and uncertainties that characterised transition into practice [7]. Brennan et al [26] also emphasized that new health professionals should cultivate a ‘doing, not observing’ attitude during transition into practice.

### Theme 4: Mediating processes

This theme captured the strategies employed by new health professionals to change or manage challenges they encountered during transition into practice. Four sub-themes emerged, namely *learning as an ongoing process, establishing supportive contacts, asking for help,* and *effective time management.*

#### Learning as an ongoing process

Research emphasized the importance of new health professionals recognising that professional competence comes through continuous learning and experience [7, 17, 33]. New health professionals should not expect themselves to know everything when transitioning into practice, rather, they should view their knowledge and skills within the confines of being a new health professional [18]. With this mindset, new health professionals were advised to strive towards professional competence through personal reading [4, 22], revisiting lecture notes [4], taking continuing education courses [6], learning from the mistakes they make [25], creating informal learning culture together with peers [36] and observing and learning from experienced senior colleagues [25].

#### Establishing supportive contacts

The sources reviewed suggested that new health professionals seek to improve their clinical competence through establishing contacts with significant others. New health professionals reported that their peers assisted in alleviating the stressors of transition [4, 6, 11, 16–18, 23, 25, 31]. New health professionals established meaningful interactions with peers through peer support meetings [5, 25], study groups [5], networking [6, 17] and peer debriefing sessions [5, 22]. Other supportive contacts included previous lecturers [4], senior colleagues [5, 6] and fellow health professionals [7, 17, 30]. New health professionals reported that ensuring meaningful personal and social lives helped alleviate transition stressors [17, 29, 30]. Furthermore, healthy interprofessional relationship with other members of the multidisciplinary team was emphasized as a positive factor in transition [28, 30].

#### Asking for help

New health professionals resorted to ‘asking for help’ when they did not know what to do [36] and sought supervision when confronted with new situations [4, 6]. In situations where there were no mentors and supervisors, new health professionals sought remote mentors and coaches [6]. Listening and regularly asking questions were also emphasized as coping strategies to ameliorate the challenges of transition [4, 22].

#### Effective time management

Effective time management strategies were found to help alleviate some challenges of transition [6, 7]. New occupational therapy graduates reported that managing their time well enabled them to deal with overwhelming work schedules, prevented having to work overtime and allowed time for meaningful personal and social lives [6].

## Conclusions

The sources included in our review highlighted numerous challenges faced by new health professionals during their transition into practice and support strategies used to ameliorate the difficulties experienced. The coping strategies employed by new health professionals in making a successful transition were included in Table 3. The review confirmed the importance of tried and tested strategies; yet, highlighted the importance of making these strategies accessible. Considerations for accessibility included availability, quality, timing and format of such strategies.

**Table 3:** Identified barriers and proposed strategies

Barriers	Strategies/Facilitators
**Systems and structures**	
1. Complex workplace systems: Complex hierarchies, administrative processes workplace politics and organisational dynamics [4, 6, 14, 16, 33, 36]. 2. Stereotyped operational cultures [15, 17] 3. Complex and overwhelming work-related responsibilities [3, 4, 15, 28, 34].. 4. Unrealistically high expectations [3, 13, 25, 26, 29]. 5. Lack of recognition and respect [5, 17, 29, 30, 33]	1. Sufficient orientation programmes [11, 13, 16, 31, 32]. 2. Preceptorship programmes [13, 28, 31]. 3. Mentoring programmes [5, 16, 22]. 4. Support from experienced senior colleagues [4, 5, 11, 16, 23, 30, 31]. 5. Peer group [4]. 6. Constructive feedback on performance from seniors, colleagues and clients [32, 34]. 7. Effective supervision [6, 7, 11, 13].
**Personal capacities**	
1. Reduced perception of self-competence and unreadiness for practice [4, 6, 7, 18–22, 26, 27, 31–33]. 2. Overwhelming feeling of inadequacy [5, 7, 23, 29] 3. Reduced self-confidence [4, 10, 11, 17, 21, 22, 24, 32]	1. Excitement in acquiring new skills and growing in their professions [3, 5]. 2. Motivation by associating their role to spiritual benefits [17].
**Professional competences**	
1. Disparity between academics and actual practice [3, 4, 16, 21, 29]. 2. High levels of tension, anxiety and nervousness upon entering the world of practice [6, 7, 20, 25–27, 29, 32, 33]. 3. Inability to manage stressful emotional work-related situations [13, 20, 23]. 4. Role confusion [6, 13, 29] 5. Inadequacy in clinical practice skills such as communication skills [10, 22, 24], organisational and management skills [16, 22], clinical decision-making skills [5, 22] and skills required for specific practice areas [6, 16, 29]. 6. Insufficient practical and clinical exposure in training [4–6, 21].	1. Prior clinical placement experiences helps ameliorate the stress and uncertainties that characterised transition into practice [4, 5, 7, 24]. 2. Cultivating a ‘doing, not observing’ attitude during transition into practice [26].
**Mediating processes (*Strategies/Facilitators for this theme apply to all three themes above*)**	
	1. Recognition that competence comes through continuous learning [7, 17, 33]. Through a. Personal reading [4, 22]. b. Revisiting lecture notes [4]. c. Taking continuing education courses [6]. d. Learning from the mistakes they make [25] e. Creating informal learning culture together with peers [36] f. Observing and learning from experienced senior colleagues [25]. 2. Establishing supportive contacts [4, 6, 11, 16–18, 23, 25, 31]. Through: a. Peer support meetings [5, 25], b. Study groups [5], c. Networking [6, 17] d. Peer debriefing sessions [5, 22]. e. Previous lecturers [4], f. Senior colleagues [5, 6] g. Fellow health professionals [7, 17, 30]. 3. Meaningful personal and social lives help alleviate transition stressors [17, 29, 30]. 4. healthy intra and interprofessional relationship [28, 30]. 5. Asking for help when uncertain [4, 6, 36]. 6. Seeking remote mentors and coaches [6]. 7. Effective listening and regularly asking questions [4, 22]. 8. Effective time management [6, 7].

Orientation programmes are needed. These should include information on systems and procedures, presented in a format that is easily accessible to new generation learners, and detailed and comprehensible enough to deal with challenges that cause unnecessary anxiety. Rather than once-off orientation programmes, modes of delivery and timing should be considered to ensure availability of information when most needed. Additionally, cumbersome and irrelevant systems and structures should be modified to make navigation easier. This may go a long way to improve accessibility and productivity.

In addition to ongoing support from line managers, new health professionals benefit from mentor and peer support. Orientation programmes should encourage new health professionals to request support or supervision from senior colleagues. Conversely, senior colleagues should maintain a good professional relationship with new health professionals and accord them due respect and recognition. This will make it easy for new graduates to approach senior colleagues for professional assistance.

Support programmes are required to assist new health professionals with closing the gap between learning and practice. Education programmes should aim at increasing the practical experiences of students to foster development of skills such as communication skills, clinical decision-making skills, management, organisational skills and time management strategies. However, care should be taken to normalize the gap between competencies new health professionals bring to the field and the clinical expectations they face. This should be done in such a way as to remove the expectation that new health professionals should already have all the competencies required to work effectively, thus promoting engagement in continued professional development activities as a virtue.

Ongoing learning should be an explicit expectation for all health professionals. Continued professional development activities should be readily available and attendance of these encouraged. Line managers and mentors of new health professionals should be sensitised to the fact that certain competencies can only be acquired during the transition into practice. This should be done in such a way as to empower them to support the learning that is still required.

Collaborative formulation of development plans and guided navigation of available support resources should be encouraged. As part of support or mentoring programmes, more experienced health professionals could guide new health professionals to reflect on areas of development and explore and identify personal strengths and the environmental resources that can be used to meet the demands of their new role. Supervisors, mentors or senior colleagues therefore assist new health professionals to identify areas of development, set goals and develop a plan of action with regard to specific knowledge and skill set they are in need of acquiring or further developing. This could assist in increasing new health professionals’ belief in their personal capabilities.

We recommend both formal and informal systems fostering the creation of support networks which ideally should be quality assured and included in performance appraisal structures. Additionally, new health professionals should utilise supportive contacts such as peer support meetings, study groups, peer debriefing sessions and previous lecturers and educators. New health professionals who find themselves in settings without supervisors can seek remote mentors and coaches who can offer long-hand supervision using virtual means. New health professionals should be open-minded and be willing to ask questions and seek help.

## Data Availability

All data generated or analysed during this review are included in this published article.

## Abbreviations

PRISMA: Preferred Reporting Items for Systematic Reviews and Meta-Analysis
CINAHL: Cumulative Index of Nursing and Allied Health Literature
